# Supplementary material for: Mapping nucleolus-associated chromatin interactions using nucleolus Hi-C reveals pattern of heterochromatin interactions
Source: Nat Commun. 2023 Jan 21;14:350. doi: 10.1038/s41467-023-36021-1 (PMC9867699; doi:10.1038/s41467-023-36021-1)
Supplement: Supplementary file 1 — Supplementary Information [file 41467_2023_36021_MOESM1_ESM.pdf]

Mapping Nucleolus-associated Chromatin Interactions Using Nucleolus Hi-C Reveals Pattern of Heterochromatin Interactions

SUPPLEMENTARY INFORMATION

Supplementary Figures and Legends

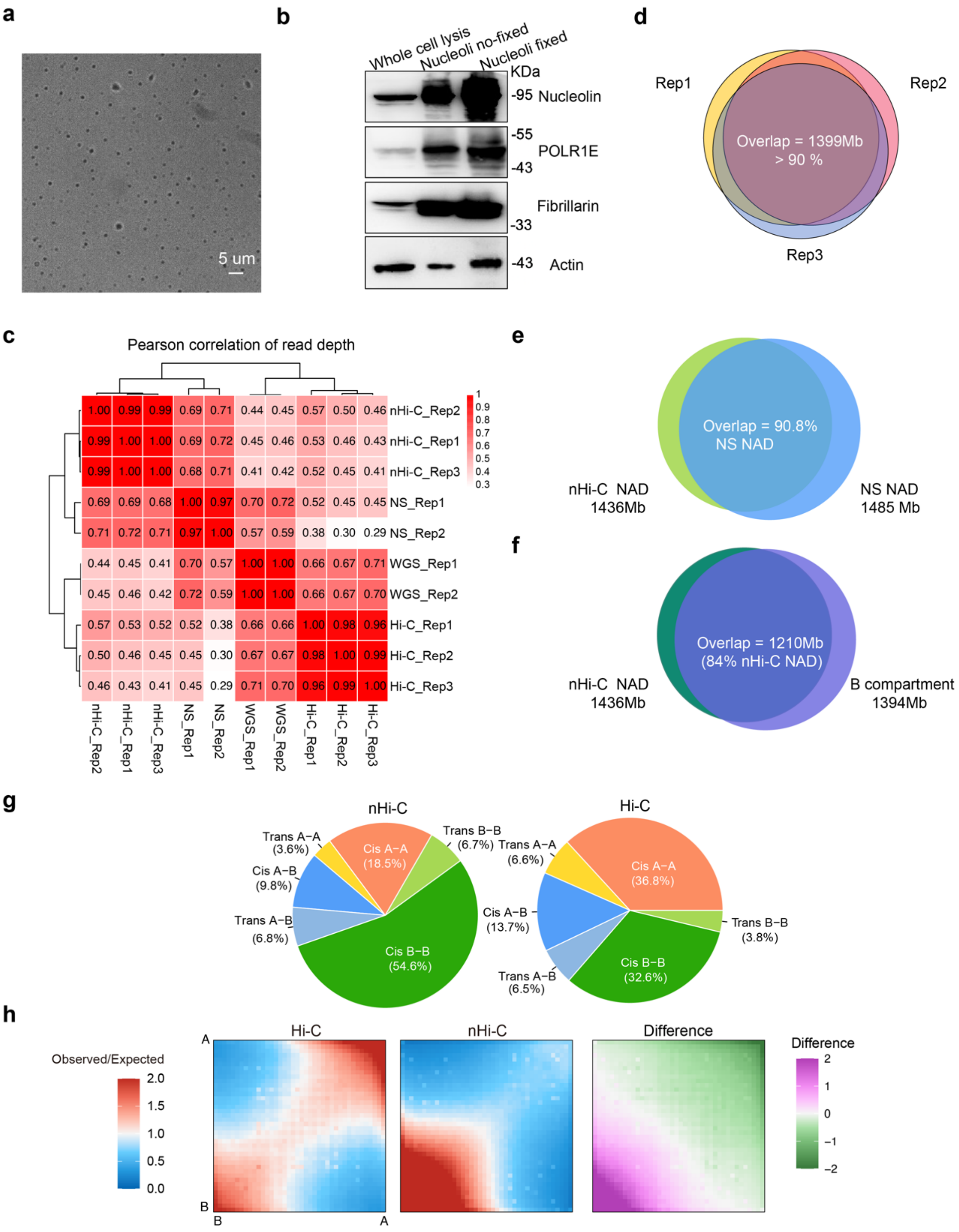

Figure S1. Quality assessment of nHi-C experiments and data in HeLa cells.

a Isolated nucleoli under microscopy (n = 3 biologically independent samples).

b Western blotting of nucleolar proteins from whole cells and isolated nucleoli (n = 3 per group). Uncropped gels are provided in the Source Data file. **c**

Correlation heatmap between sequencing data. NS: nucleolus sequencing. '_Rep' stands for different biological replicates. **d** Overlap of nHi-C NADs in different replicates. **e** Overlap between nHi-C NADs and NS-seq NADs. **f** Overlap between nHi-C NADs and B compartments. **g** Percentage of *cis*/*trans* A-A, A-B, and B-B compartment interactions captured by nHi-C and *in situ* Hi-C in HeLa cells. **h** Saddle plot of interactions captured by nHi-C and *in situ* Hi-C.

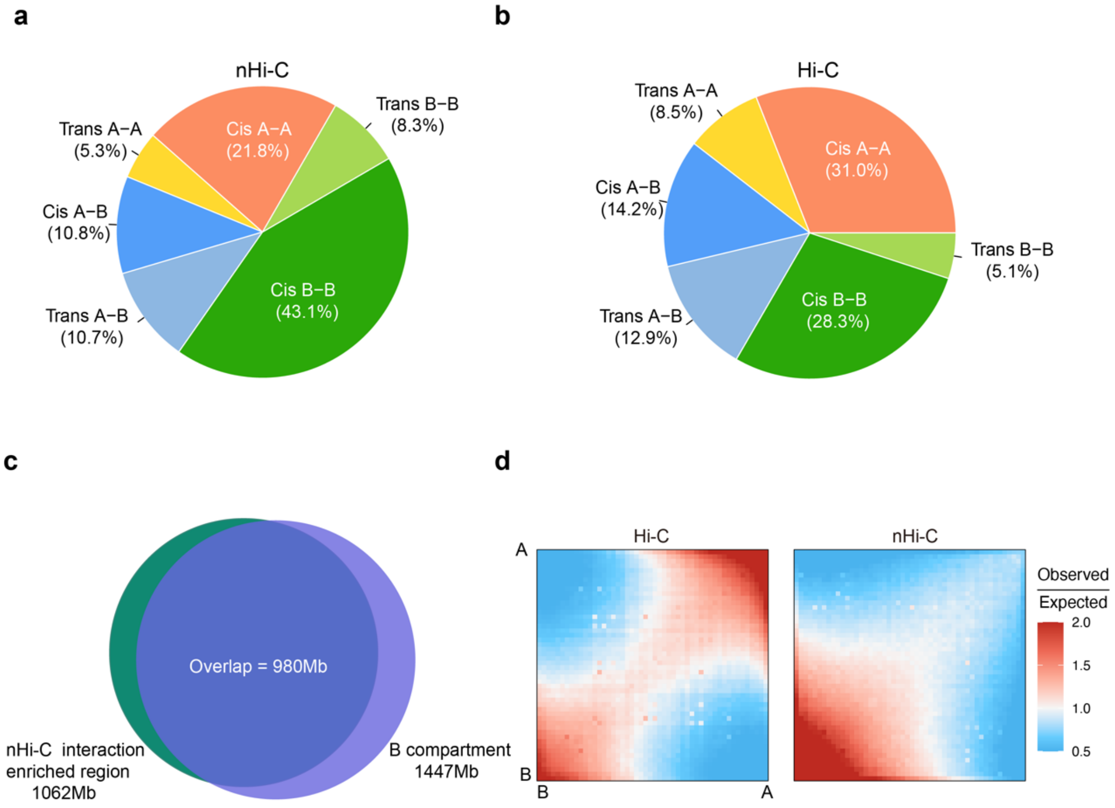

Figure S2. Quality assessment of nHi-C experiments and data in U2OS cells.

a-b Percentage of *cis*/*trans* A-A, A-B, and B-B compartment interactions captured by nHi-C and *in situ* Hi-C in U2OS cells. **c** Overlap between nHi-C

interaction-enriched regions and B compartments. **d** Saddle plot of interactions captured by nHi-C and *in situ* Hi-C.

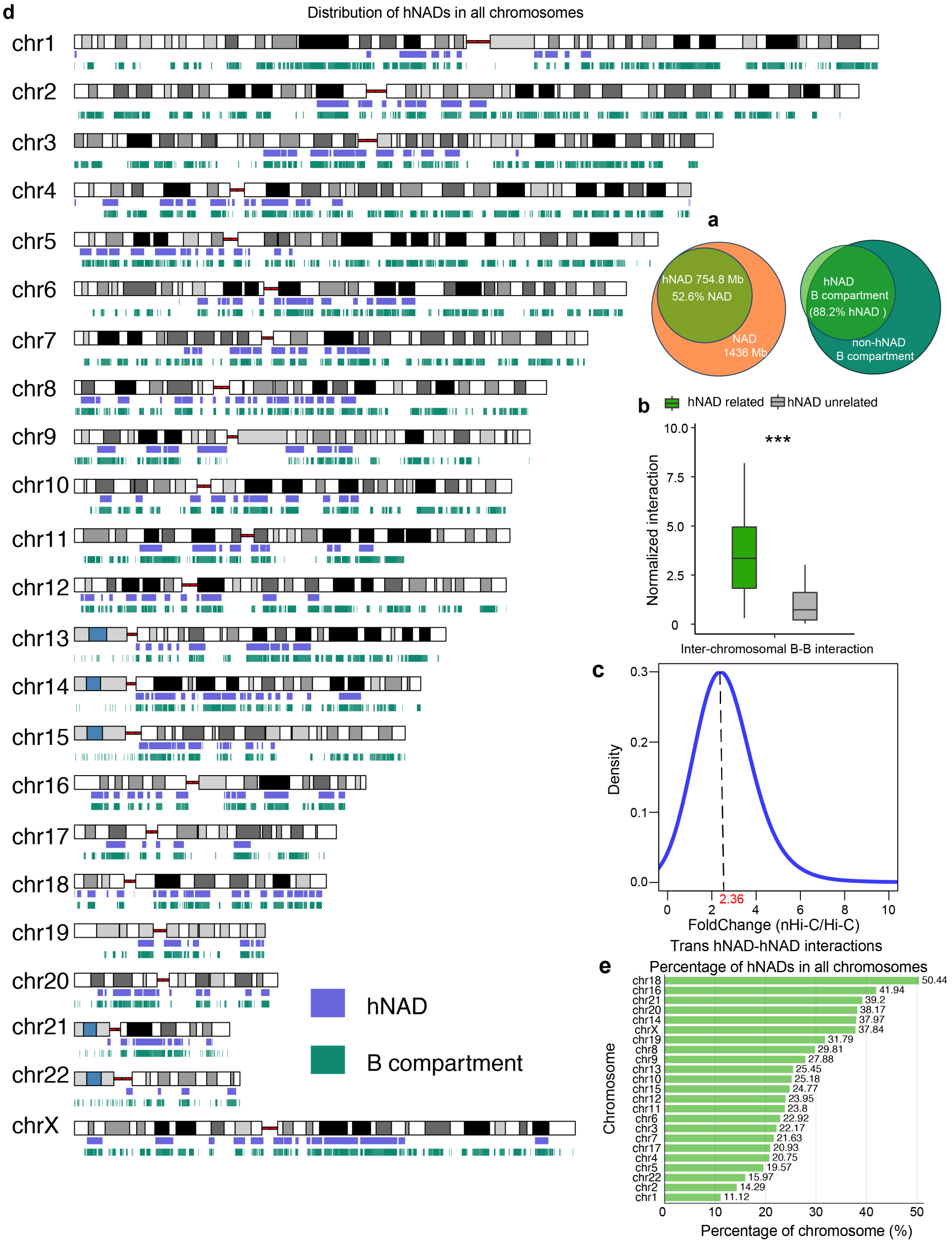

Figure S3. Genomic distribution of hNADs.

a hNADs account for 52.6% of all NADs, and 88.2% of hNADs overlap with B compartments. **b** Comparison of hNAD-related and unrelated inter-

chromosomal B-B interactions. In box-plots, center line stands for median; box limits are 25th and 75th percentiles; whiskers are min to max. n = 23 chromosomes. Statistically significant differences are indicated, and were calculated with two-sided Wilcoxon test. ***p < 0.001. **c** Density distribution of the fold change nHi-C/Hi-C in *trans* hNAD-hNAD interactions. **d-e** Distribution of hNADs in all chromosomes.

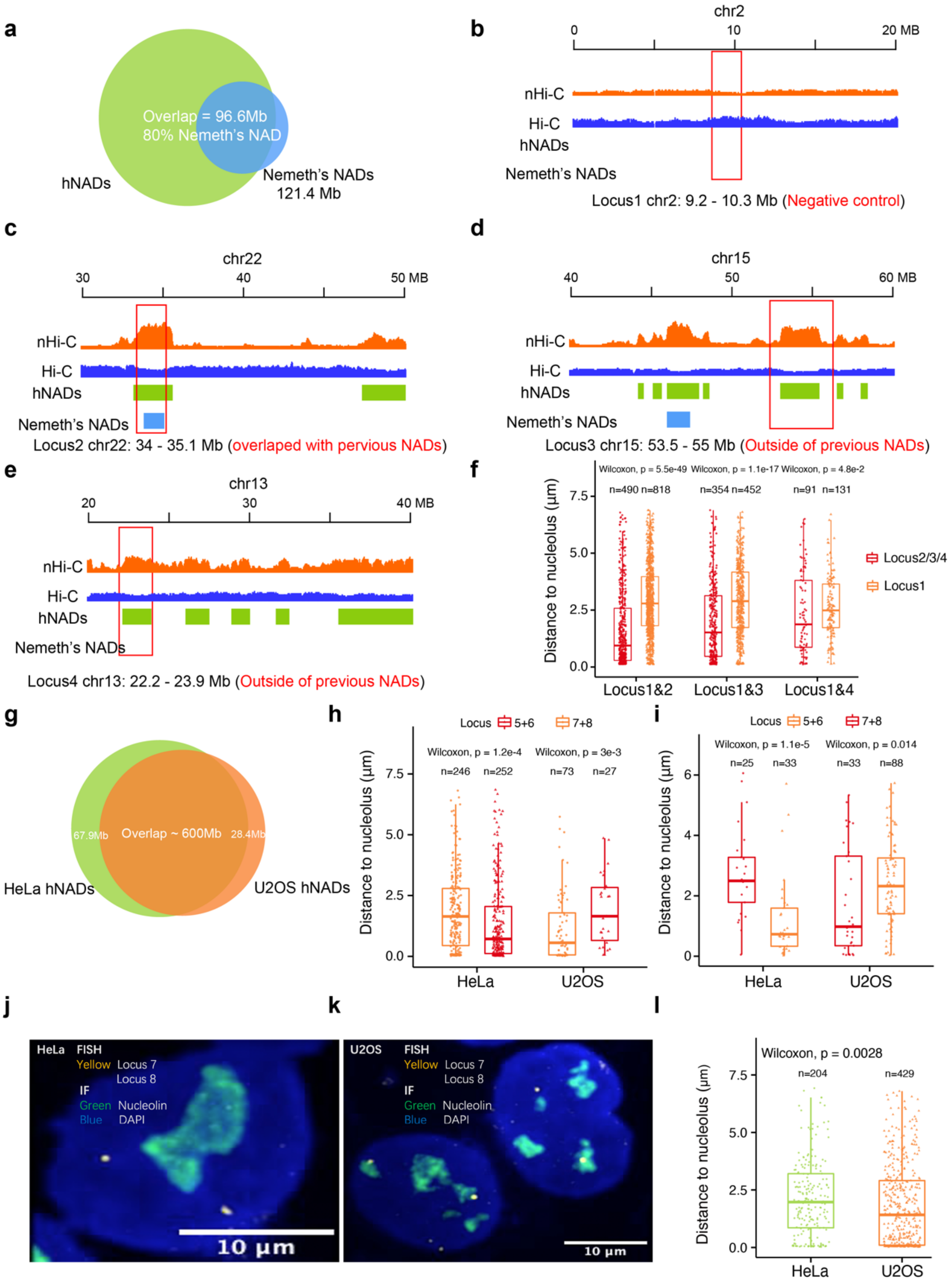

Figure S4. Chosen hNADs for DNA-FISH validation.

a Overlap between hNADs and NADs identified by Nemeth et al. **b-e** Track plot of genome regions chosen to perform DNA-FISH. **f** Distance between hNADs/negative control Locus 1-4 and nucleolus in HeLa cells. **g** Overlap

between hNADs in HeLa cells and U2OS cells. **h-i** Distance between U2OS-specific hNADs Locus 7 and 8/HeLa-specific hNADs Locus 5 and 6 and nucleolus in HeLa and U2OS cells. **j-k** Representative images of U2OS-specific hNADs' Oligopaint FISH probes Locus 7 and 8 in HeLa and U2OS cells (n = 5 biologically independent samples). **l** Distance between U2OS-specific hNADs Locus 7 and 8 and nucleolus in HeLa and U2OS cells. In all box-plots (f, h-i, l), center line stands for median; box limits are 25th and 75th percentiles; whiskers are min to max. The number of 'n' indicates the total number of cells imaged in independent experiments. Statistically significant differences are indicated, and were calculated with two-sided Wilcoxon test.

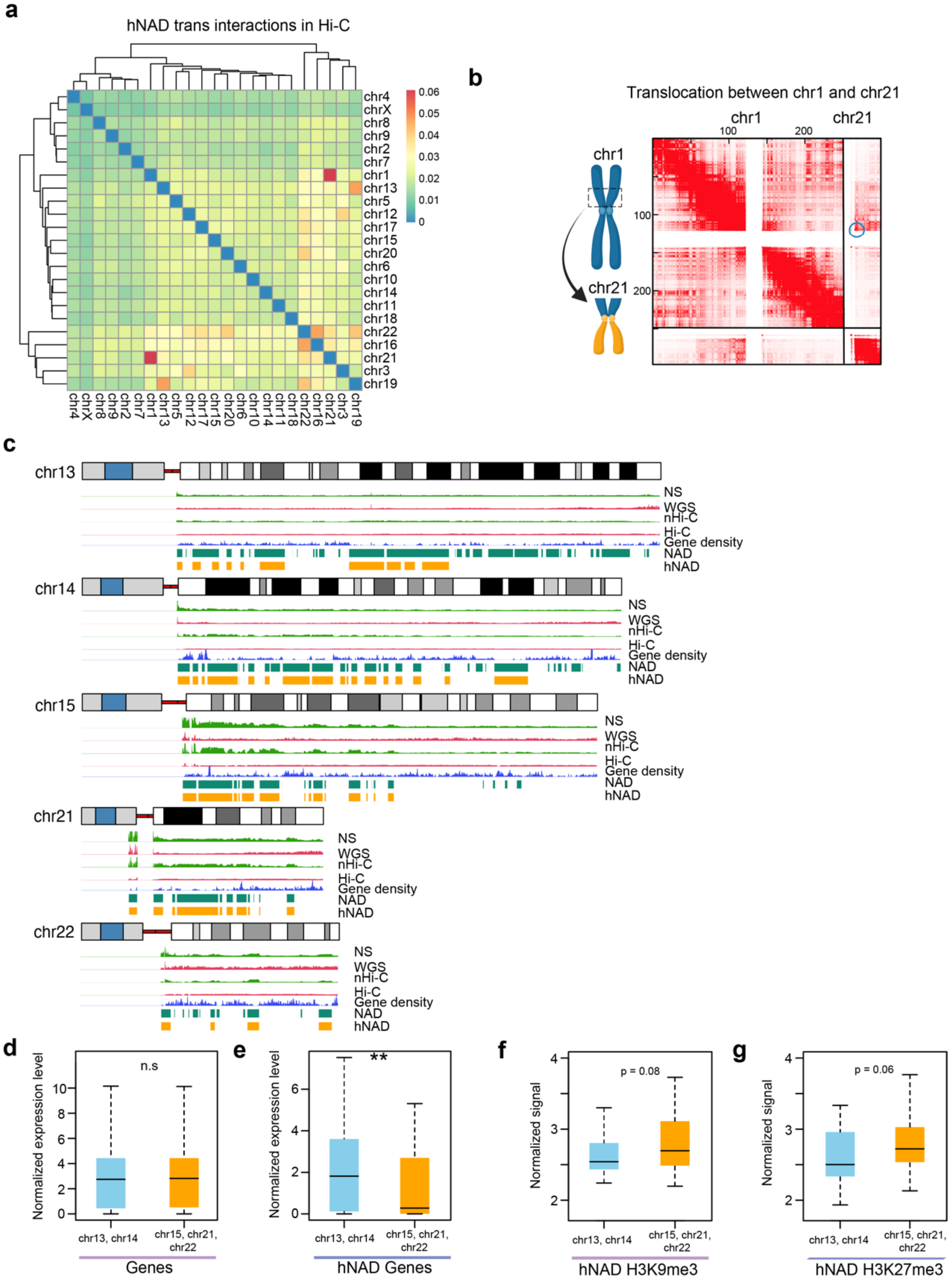

Figure S5. Interactions among the five NOR-bearing chromosomes.

a Chromosome clustering results of hNAD *trans* interactions captured by *in situ* Hi-C. **b** Interaction heatmap of chromosomes 1 and 21. **c** Coverage of nucleolus-seq, WGS, nHi-C, and Hi-C in NOR-bearing chromosomes. **d** Gene

expression levels in NOR-bearing chromosomes. n = 1263 genes on chr13/chr14, n = 1768 genes on chr15/chr21/chr22. **e** Expression levels of hNAD genes in NOR-bearing chromosomes. n = 246 hNAD genes on chr13/chr14, n = 312 hNAD genes on chr15/chr21/chr22. **f-g** H3K9me3 (f) and H3K27me3 (g) signals at hNADs located on NOR-bearing chromosomes. n = 28 hNADs in chr13/chr14, n = 31 hNADs on chr15/chr21/chr22. In all box-plots (d-g), center line stands for median; box limits are 25th and 75th percentiles; whiskers are min to max. Statistically significant differences are indicated, and were calculated with two-sided Wilcoxon test. **p < 0.01.

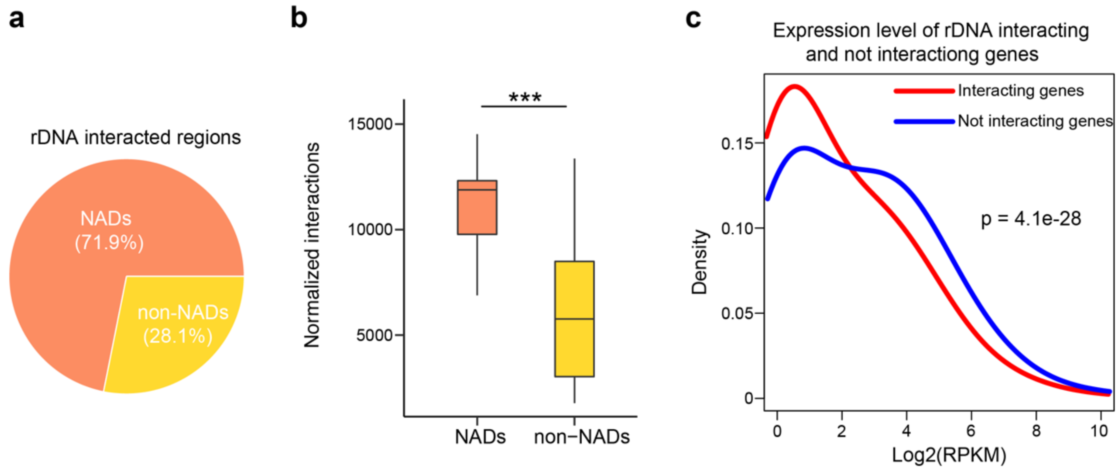

Figure S6. **rDNA prefers to form interactions with NADs.**

a Percentage of rDNA-interacting regions located in NAD and non-NAD regions. **b** rDNA-related interactions are significantly enriched in NAD regions. In box-plots, center line stands for median; box limits are 25th and 75th percentiles; whiskers are min to max. n = 23 chromosomes. Statistically significant differences are indicated, and were calculated with two-sided Wilcoxon test. ***p < 0.001. **c** rDNA-interacting genes have significantly lower expression

levels than other genes. Statistically significant differences are indicated, and were calculated with two-sided Wilcoxon test.

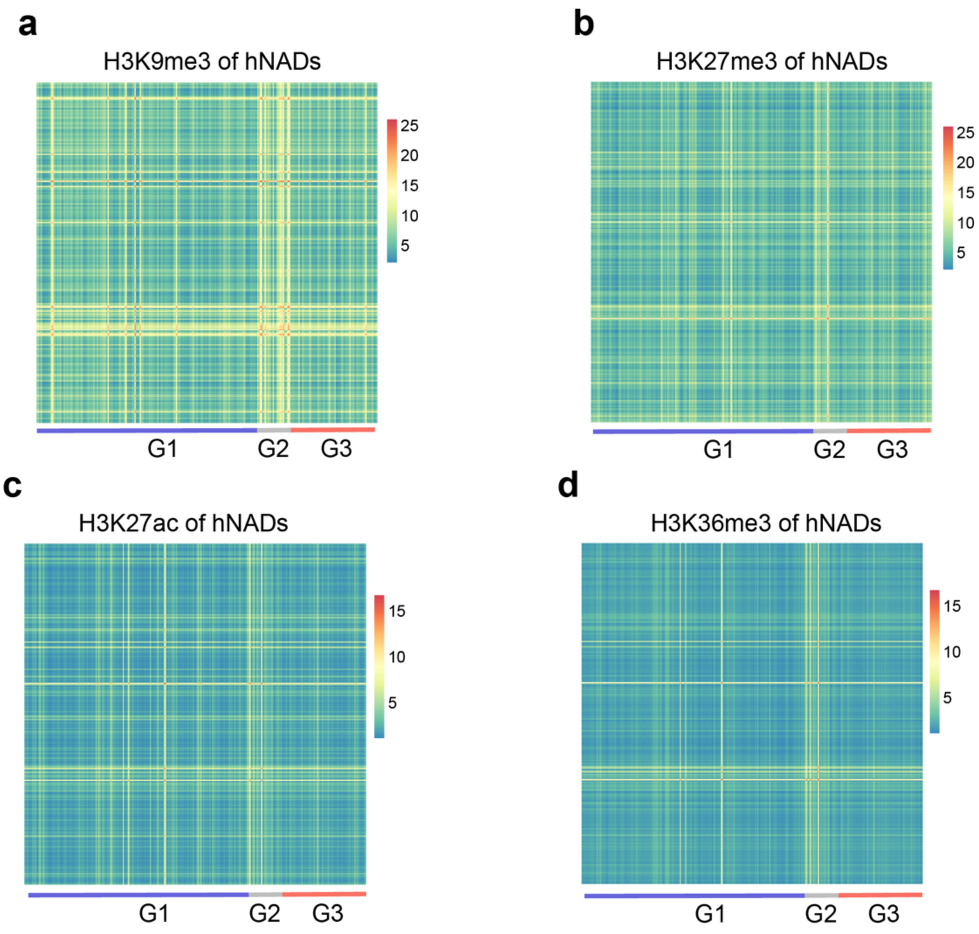

Figure S7. **Epigenetic modifications of three hNAD subgroups.**

a-d Histone modification levels in the G1, G2, and G3 hNAD regions. a H3K9me3, b H3K27me3, c H3K27ac, d H3K36me3.

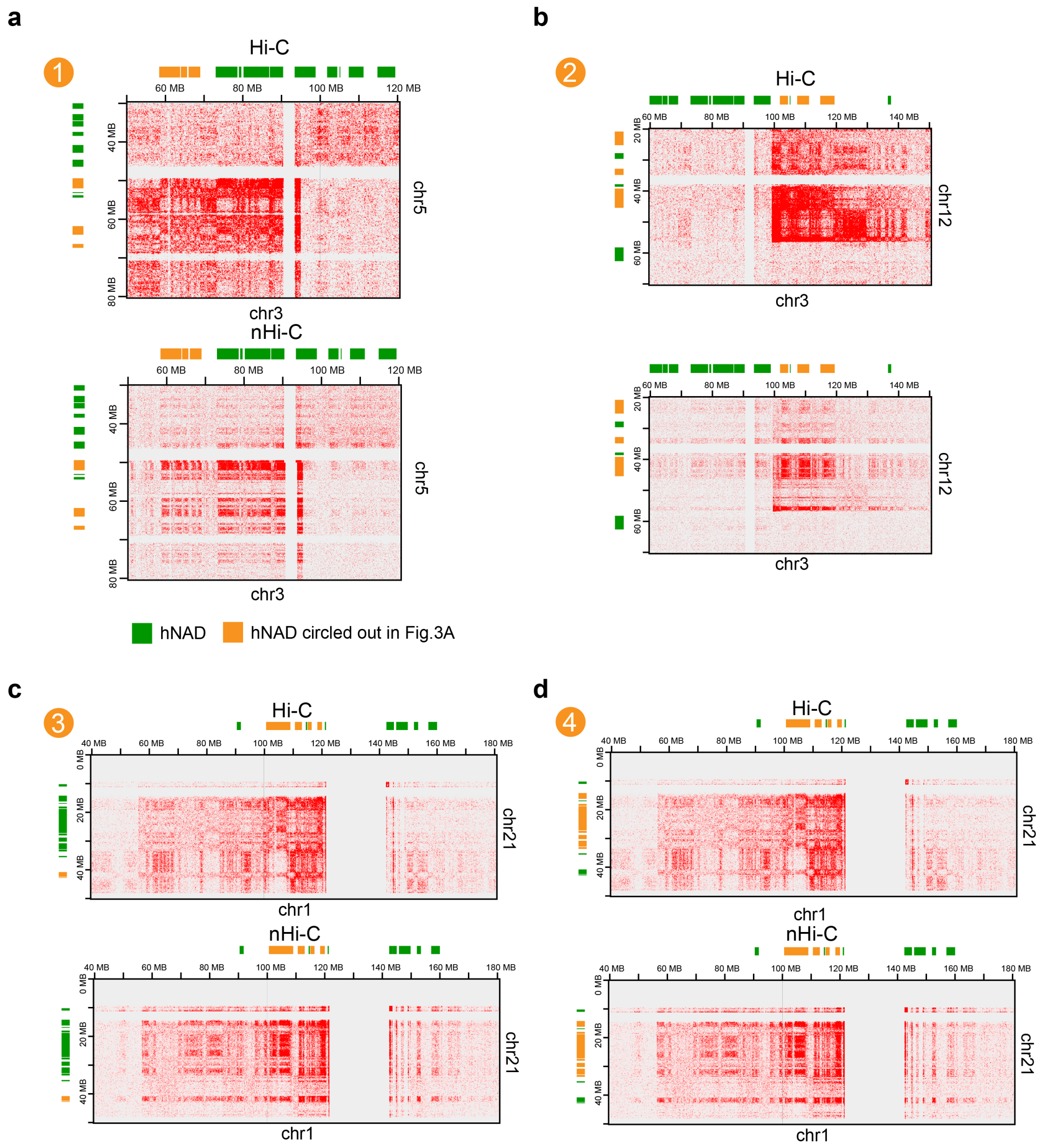

Figure S8. **hNAD-related chromosome translocations.**

a-d Labeled hotspots in Fig. 3A form butterfly style *trans* interaction heatmaps and are all identified as translocations in both Hi-C and nHi-C.

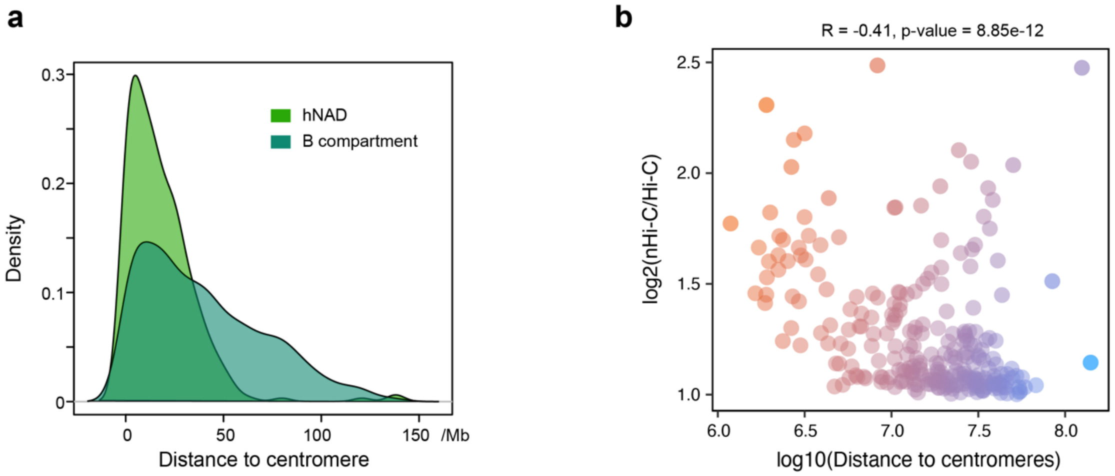

Figure S9. **hNADs show positive connections with centromeres.**

a The distance of hNAD and B compartment regions from centromeres. **b** Scatter plot of the distance between hNADs and centromeres x axis and the hNAD signal ratio y axis. Statistically significant differences are indicated, and were calculated with two-sided Pearson correlation test.

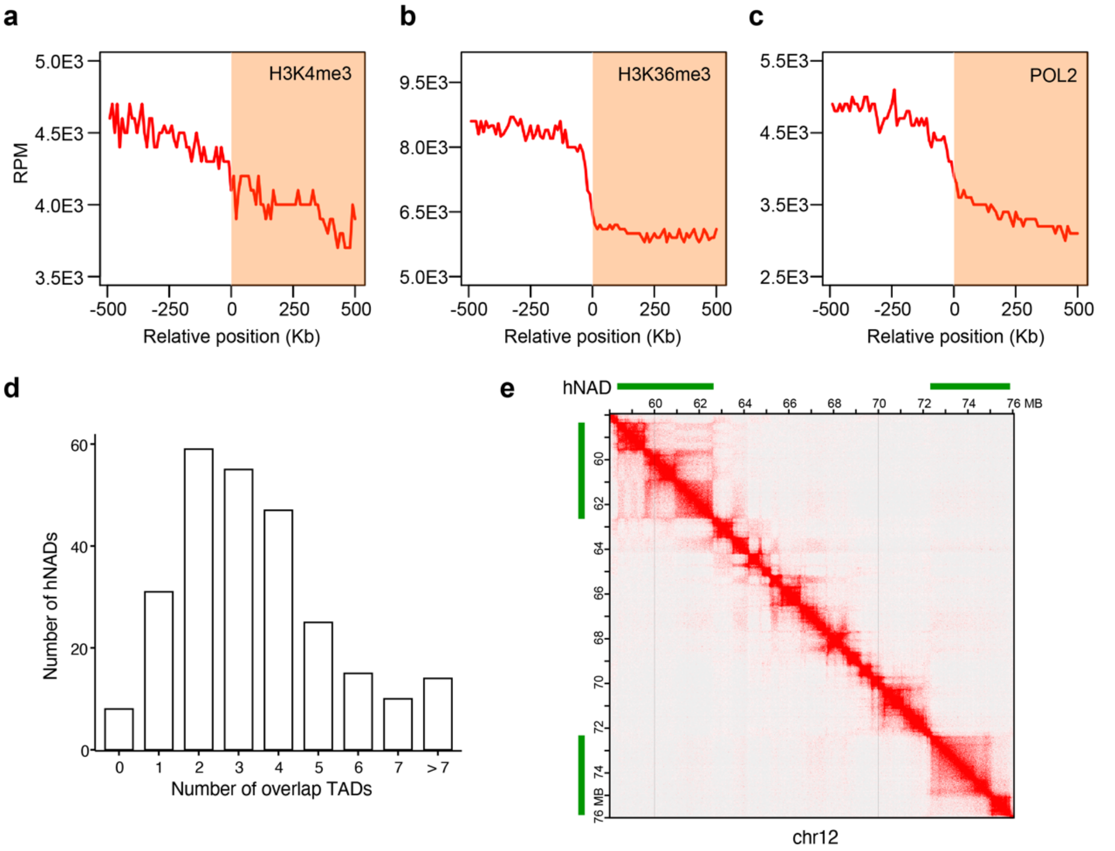

Figure S10. Active histone modifications and RNA polymerase II binding signal at hNAD boundaries.

a H3K4me3, **b** H3K36me3, **c** RNA polymerase II. Orange region indicates hNAD regions. **d** Distribution of hNADs overlapping with TADs. **e** An example of overlap between hNADs and TADs.

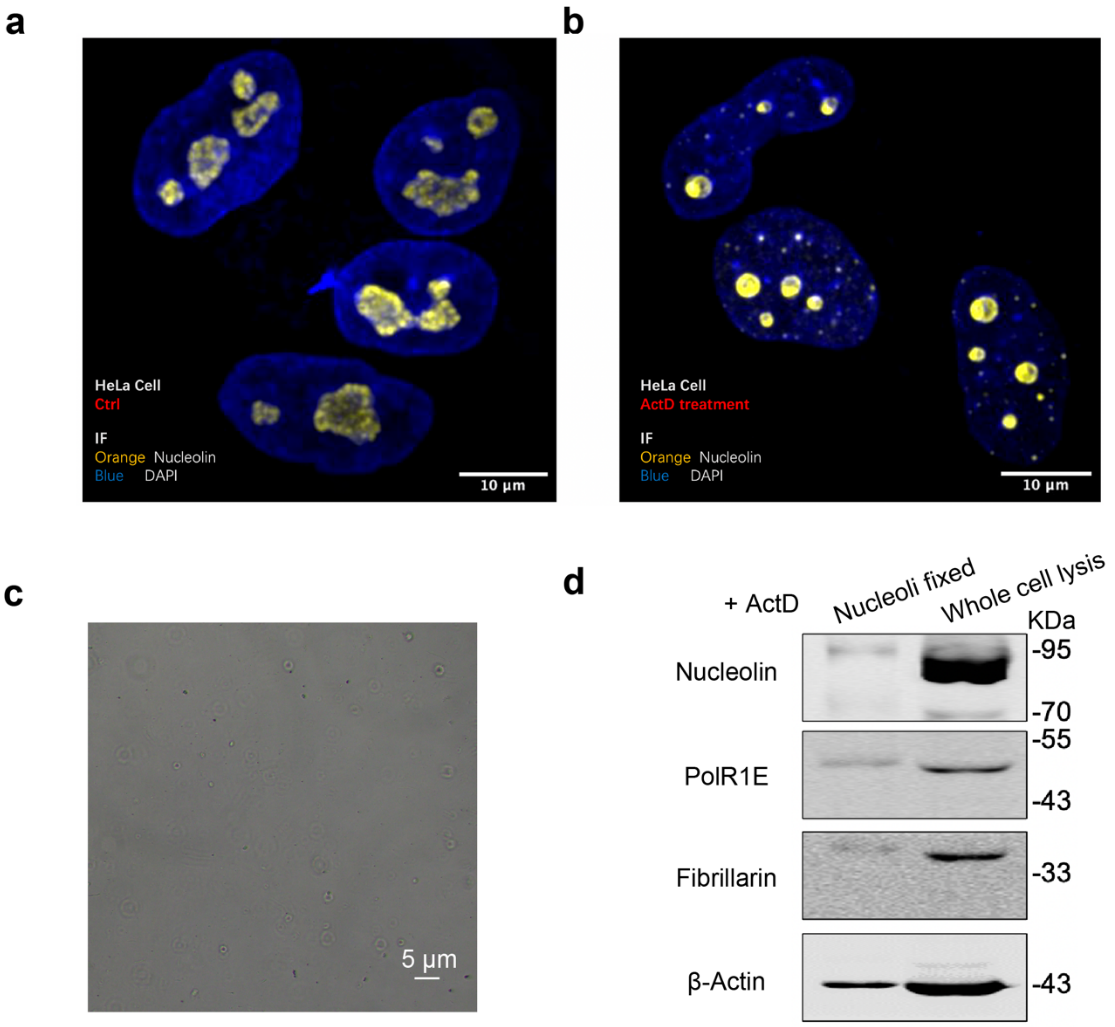

Figure S11. Nucleolus isolation after ActD treatment.

a-b Immunostaining results of nucleolin in ActD non-treat (a) and ActD treated HeLa cells (b) (n = 3 biologically independent samples). **c** Isolated nucleoli after ActD treatment under microscopy (n = 3 biologically independent samples). **d** Western blotting of nucleolar proteins from isolated nucleoli after ActD

treatment (n = 2 per group). Uncropped gels are provided in the Source Data file.

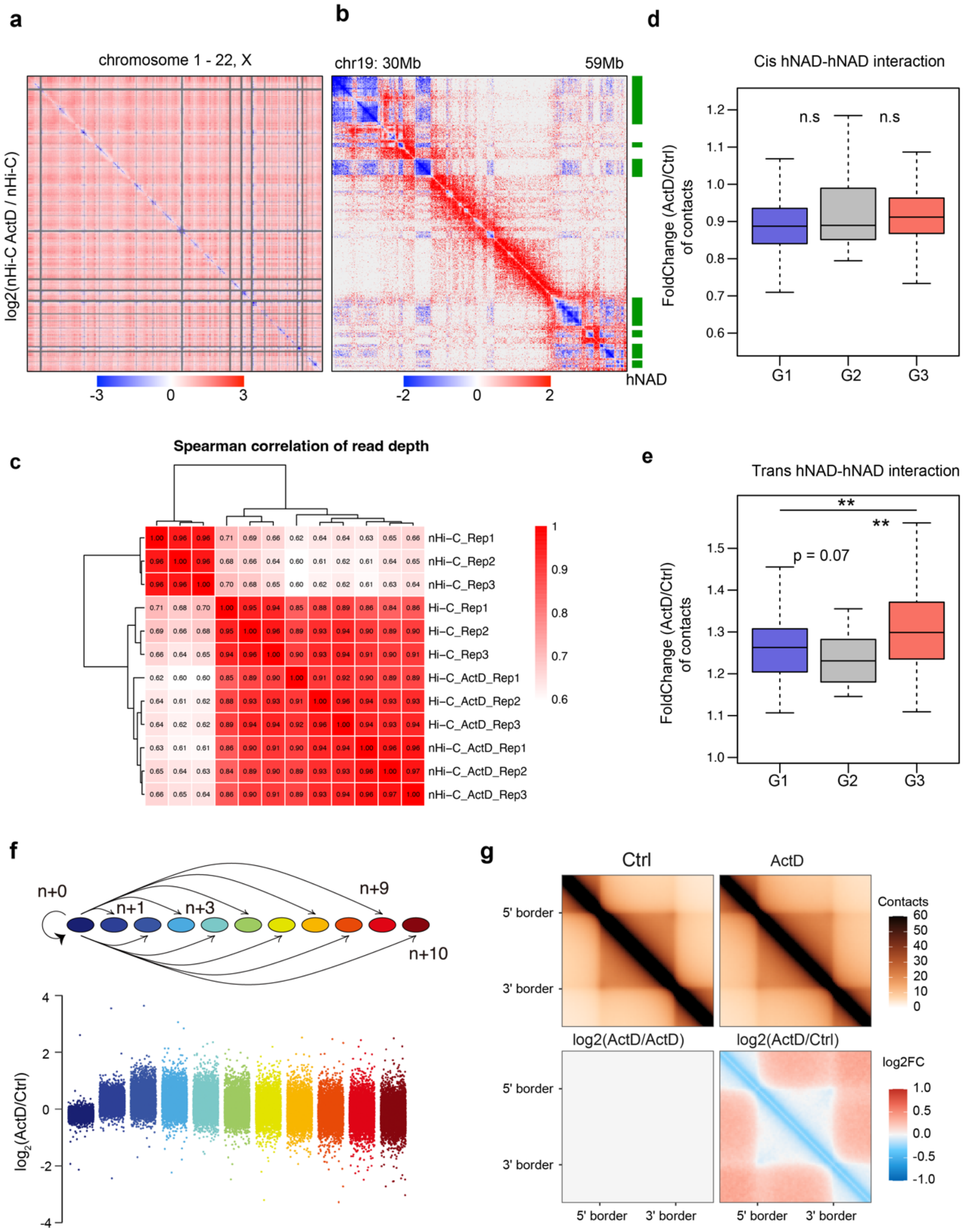

Figure S12. ActD treatment induces genome reorganization in HeLa cells. **a** Comparison $\log_2$nHi-C/Hi-C heatmap of all chromosomes. **b** An example of interaction changes at hNADs. **c** Correlation of read depth in Hi-C and nHi-C

data. '_Rep' stands for different biological replicates. **d-e** *Cis* (d) and *trans* (e) hNAD-hNAD interaction changes at G1, G2, and G3 hNADs. In box-plots, center line stands for median; box limits are 25th and 75th percentiles; whiskers are min to max. n = 177 (G1), n = 22 (G2), n = 65 (G3). Statistically significant differences are indicated, and were calculated with two-sided Wilcoxon test. **p < 0.01. **f** Changes in intra- and inter-TAD interactions after ActD treatment. **g** ATA plot of intra- and inter-TAD interaction changes.
