# Supplementary material for: Mapping nucleolus-associated chromatin interactions using nucleolus Hi-C reveals pattern of heterochromatin interactions
Source: Nat Commun. 2023 Jan 21;14:350. doi: 10.1038/s41467-023-36021-1 (PMC9867699; doi:10.1038/s41467-023-36021-1)
Supplement: Supplementary file 3 — Description of additional Supplementary File [file 41467_2023_36021_MOESM3_ESM.pdf]

Descriptions of additional supplementary Files

Supplementary Data1: Statistical information of Hi-C and nucleolus Hi-C libraries.

Supplementary Data2: Statistical information of whole genome sequencing and nucleolus sequencing libraries.

Supplementary Data3: Genomic location of 264 hNADs.

Supplementary Data4: Sequence of oligo pools in FISH experiments.

Supplementary Data5: Sequence of secondary probe sets in FISH experiments.

Supplementary Movie1-3: 3D FISH results of Fig. 2c-e.

Supplementary Movie4: 3D FISH results of Fig. 2h.

Supplementary Movie5-7: 3D FISH results of Fig. 2k-m.

Supplementary Movie8-9: 3D FISH results of Supplementary Fig. 4j-k.

Supplementary Movie10-11: 3D FISH results of Supplementary Fig. 11a-b.
