# Supplementary material for: Mapping nucleolus-associated chromatin interactions using nucleolus Hi-C reveals pattern of heterochromatin interactions
Source: Nat Commun. 2023 Jan 21;14:350. doi: 10.1038/s41467-023-36021-1 (PMC9867699; doi:10.1038/s41467-023-36021-1)
Supplement: Supplementary file 6 — Reporting Summary [file 41467_2023_36021_MOESM6_ESM.pdf]

nature portfolio

Corresponding author(s): Cheng Li, Tingting Li

Last updated by author(s): Dec 22, 2022

Reporting Summary

Nature Portfolio wishes to improve the reproducibility of the work that we publish. This form provides structure for consistency and transparency in reporting. For further information on Nature Portfolio policies, see our Editorial Policies and the Editorial Policy Checklist.

Statistics

For all statistical analyses, confirm that the following items are present in the figure legend, table legend, main text, or Methods section.

n/a	Confirmed	
☐	☒	The exact sample size (*n*) for each experimental group/condition, given as a discrete number and unit of measurement
☐	☒	A statement on whether measurements were taken from distinct samples or whether the same sample was measured repeatedly
☐	☒	The statistical test(s) used AND whether they are one- or two-sided<br>*Only common tests should be described solely by name; describe more complex techniques in the Methods section.*
☒	☐	A description of all covariates tested
☐	☒	A description of any assumptions or corrections, such as tests of normality and adjustment for multiple comparisons
☐	☒	A full description of the statistical parameters including central tendency (e.g. means) or other basic estimates (e.g. regression coefficient) AND variation (e.g. standard deviation) or associated estimates of uncertainty (e.g. confidence intervals)
☐	☒	For null hypothesis testing, the test statistic (e.g. *F*, *t*, *r*) with confidence intervals, effect sizes, degrees of freedom and *P* value noted<br>*Give P values as exact values whenever suitable.*
☒	☐	For Bayesian analysis, information on the choice of priors and Markov chain Monte Carlo settings
☐	☒	For hierarchical and complex designs, identification of the appropriate level for tests and full reporting of outcomes
☐	☒	Estimates of effect sizes (e.g. Cohen's *d*, Pearson's *r*), indicating how they were calculated

Our web collection on statistics for biologists contains articles on many of the points above.

Software and code

Policy information about availability of computer code

Data collection	No software was used to collect the data.
Data analysis	R (version 3.6.0), STAR (version 2.7.2b), bowtie2 (version 2.3.5), Picard (version 2.21.3), HiC-Pro (version 2.11.12), SAMtools (version 1.9), MACS2 (version 2.2.5), HiNT (veision 2.2.7), ImageJ (version 1.53), OligoMiner (version 1.0.1), ImageLab (version 4.0), MATLAB (version 2022b).<br>Python package: HTSeq (version 2.0.2)<br>R packages: HiTC (version 1.38.0), GENOVA (version 1.0).<br>All essential codes of data analysis and figures for reproducible research are available on GitHub (https://github.com/ChengLiLab/nHi-C)

For manuscripts utilizing custom algorithms or software that are central to the research but not yet described in published literature, software must be made available to editors and reviewers. We strongly encourage code deposition in a community repository (e.g. GitHub). See the Nature Portfolio guidelines for submitting code & software for further information.

Data

Policy information about availability of data

All manuscripts must include a data availability statement. This statement should provide the following information, where applicable:

- Accession codes, unique identifiers, or web links for publicly available datasets
- A description of any restrictions on data availability
- For clinical datasets or third party data, please ensure that the statement adheres to our policy

The sequencing data generated in this study have been deposited in the Gene Expression Omnibus (GEO) database under accession code GSE90003 (https://www.ncbi.nlm.nih.gov/geo/query/acc.cgi?acc=GSE90003). The histone modification and CTCF ChIP-Seq data were downloaded from ENCODE. The ENCODE IDs are: ENCFF000BAJ (https://www.encodeproject.org/experiments/ENCSR000AOA/), ENCFF000BCO (https://www.encodeproject.org/experiments/ENCSR000AOF/), ENCFF000BBG (https://www.encodeproject.org/experiments/ENCSR000AQO/), ENCFF000BBS (https://www.encodeproject.org/experiments/ENCSR000APB/), ENCFF000BCA (https://www.encodeproject.org/experiments/ENCSR000AOD/).

Human research participants

Policy information about studies involving human research participants and Sex and Gender in Research.

Reporting on sex and gender	Not relevant. This study did not involve human research participants.
Population characteristics	Not relevant. This study did not involve human research participants.
Recruitment	Not relevant. This study did not involve human research participants.
Ethics oversight	Not relevant. This study did not involve human research participants.

Note that full information on the approval of the study protocol must also be provided in the manuscript.

Field-specific reporting

Please select the one below that is the best fit for your research. If you are not sure, read the appropriate sections before making your selection.

[x] Life sciences [ ] Behavioural & social sciences [ ] Ecological, evolutionary & environmental sciences

For a reference copy of the document with all sections, see nature.com/documents/nr-reporting-summary-flat.pdf

Life sciences study design

All studies must disclose on these points even when the disclosure is negative.

Sample size	Sample sizes were selected so as to power non-parametric statistical analysis.
Data exclusions	No data were excluded in this study.
Replication	3 independent biological experiments were performed with ActD-treated and non-treated HeLa cells. Then Hi-C, nucleolus Hi-C were performed with these replicates. Results were reproducible in all of the samples analyzed. All attempts at replication were successful.
Randomization	Not relevant. This study did not involve experimental grouping.
Blinding	Not relevant. This study did not involve experimental grouping.

Reporting for specific materials, systems and methods

We require information from authors about some types of materials, experimental systems and methods used in many studies. Here, indicate whether each material, system or method listed is relevant to your study. If you are not sure if a list item applies to your research, read the appropriate section before selecting a response.

Materials & experimental systems

n/a	Involved in the study
	[x] Antibodies
	[x] Eukaryotic cell lines
[x]	Palaeontology and archaeology
[x]	Animals and other organisms
[x]	Clinical data
[x]	Dual use research of concern

Methods

n/a	Involved in the study
[x]	ChIP-seq
[x]	Flow cytometry
[x]	MRI-based neuroimaging

Antibodies

Antibodies used

Actin: Abcam, cat. ab8226
Nucleolin: Abcam, cat. ab129200
Fibrillarin: Abcam, cat. ab4566
POLR1E: Abclonal, cat. A12700
donkey anti-rabbit Alexa Fluor 488: Thermo Fisher Scientific, cat. A-21206
goat anti-rabbit Cy3: Thermo Fisher Scientific, A-10522

Validation

All antibodies used have been validated for their use in immunofluorescence.

Eukaryotic cell lines

Policy information about cell lines and Sex and Gender in Research

Cell line source(s)

HeLa (catalog number: CCL-2) and U2OS (catalog number: HTB-96) were purchased from ATCC.

Authentication

All cell lines were authenticated according to the morphology.

Mycoplasma contamination

Cell lines were not tested for mycoplasma contamination.

Commonly misidentified lines
(See ICLAC register)

No commonly misidentified cell lines were used.
